# Case Report: A rare case of a giant sporadic non-ampullary duodenal adenoma that was safely and effectively resected using hybrid endoscopic submucosal dissection

**DOI:** 10.3389/fonc.2025.1511454

**Published:** 2025-06-23

**Authors:** Jun-Jie Hou, Liang Ding, Yan-Fei Yang, Wei-Wei Li

**Affiliations:** Department of Gastroenterology, Shaoxing People’s Hospital, Shaoxing, China

**Keywords:** hybrid endoscopic submucosal dissection, sporadic non-ampullary duodenal adenomas, giant duodenal adenoma, endoscopic therapy, case report

## Abstract

**Background:**

As a rare condition, sporadic non-ampullary duodenal adenomas (SNADAs) are typically asymptomatic and diagnosed incidentally. It warrants treatment due to the high risk of malignant transformation. However, giant SNADAs, especially those treated using hybrid endoscopic submucosal dissection (ESD), are exceedingly rare, and to our knowledge, this condition has not been reported previously.

**Case presentation:**

A 69-year-old Chinese male patient was diagnosed with an incidentally discovered giant SNADA. Given the size and location and the patient’s non-invasive request, we performed hybrid ESD to completely resect a giant SNADA (4.5*2.8*1.0cm) without postoperative discomfort or complications. Final pathology confirmed a margin-negative tubulovillous adenoma with low-grade intraepithelial neoplasia.

**Conclusion:**

We present a rare case of a giant SNADA that was successfully dissected using hybrid ESD. Further, we provide a brief review, discuss the treatment protocol of this case, and provide a new perspective for the future diagnosis and treatment of a giant SNADA.

## Introduction

Duodenal adenomas are uncommon and mostly asymptomatic, often detected incidentally during endoscopic evaluation, with an estimated incidence of 0.03%–0.1% (1–3). It is generally considered that, regardless of their anatomical location and whether they are sporadic or FAP-related, duodenal adenomas carry a high risk of malignant transformation, which follows the adenoma-carcinoma sequence and is similar to the colonic adenoma-carcinoma pattern, and thus deserve treatment (4–7). Wherein, low-grade dysplasia (LGD) lesions show a low risk of progression to adenocarcinoma, while a large initial tumor size and location on the oral side of the papilla of Vater (8) warrant a careful follow-up biopsy due to the risk of progression to high-grade dysplasia (HGD). However, HGD lesions and large non-ampullary Sporadic duodenal adenomas (SDAs) ≥ 20 mm in diameter show a high risk of malignant transformation and thus should be treated immediately (9). In addition, the malignant risk of ampullary adenomas is much higher than that of non-ampullary sporadic duodenal adenomas (10, 11). Current treatment techniques for duodenal adenoma include endoscopy therapy and surgery, of which the former has the advantage of safety and less invasiveness. Endoscopic resection techniques for SDAs include snare polypectomy, argon plasma coagulation (APC) ablation, endoscopic mucosal resection (EMR), underwater EMR, and endoscopic submucosal dissection (ESD), with EMR as the primary treatment technique (1).

Here, we present a very rare case of a giant sporadic non-ampullary duodenal adenoma (SNADA) that was successfully resected using hybrid ESD. Additionally, we present a brief review and case-related discussion.

## Case presentation

**Figure 1:**
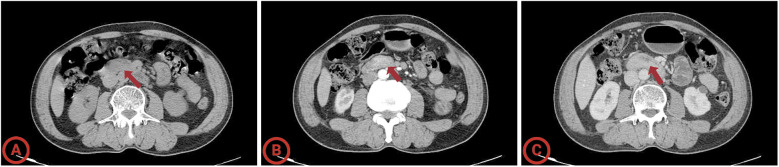
Computed tomography of the giant sporadic non-ampullary duodenal adenoma. **(A)** Arterial phase; **(B)** venous phase; **(C)** delayed scan. The average HU values of the lesion in the plain scan, arterial, and portal venous phases are 63.2 HU, 87.9 HU, and 93.1 HU.

**Figure 2:**
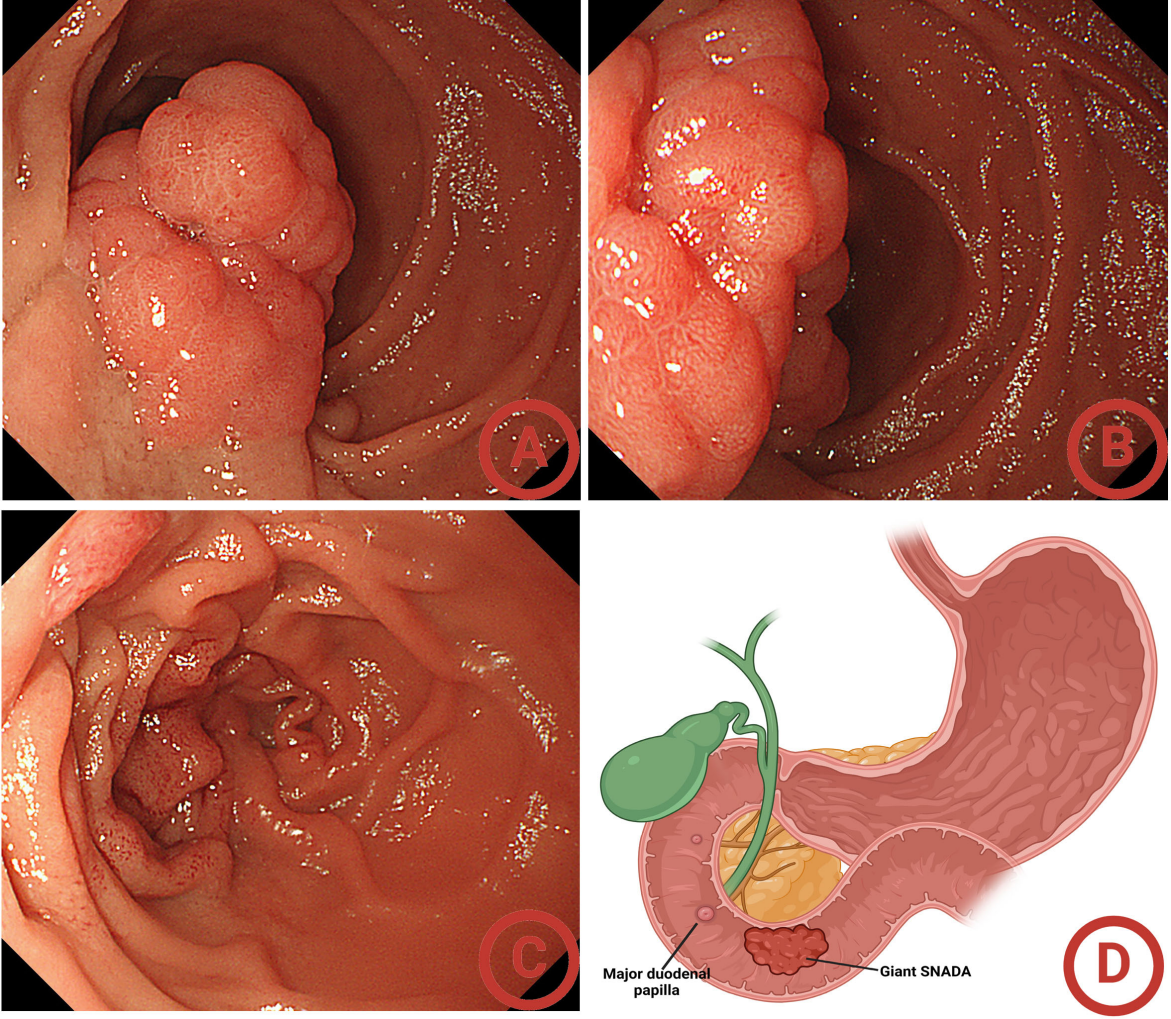
Initial gastroscopy findings. **(A)** Far-view of the giant sporadic non-ampullary duodenal adenoma (SNADA); **(B)** near-view of the giant SNADA; **(C)** confirming the location of the duodenal papilla; **(D)** the location of the giant SNADA.

A 69-year-old male patient, with a history of hypertension, visited our hospital for progressive urinary obstruction. The abdomen contrast computed tomography (CT) scan showed a soft tissue shadow in the descending duodenum, which was significantly enhanced after contrast enhancement. The position of the soft tissue shadow was slightly lowered in the portal phase, and no significant abnormalities were observed in the remaining intestinal lumen (**Figure 1**). Further transoral enteroscopy identified a giant mass of approximately 5 cm with a wide base approximately 5 to 6 cm below the duodenal papilla, the surface of which was lobulated and the glandular duct was basically present (**Figure 2**). Biopsy pathology of the mass indicated tubular adenoma, with low-grade intraepithelial neoplasia (LGIN). Combined with the enteroscopy findings and pathological findings, the duodenal mass was considered a case of giant SNADA.

Given the characteristics of the lesion and the patient’s request for surgical treatment and minimally invasive resection, we performed hybrid ESD to completely remove the adenoma and reduce the duration of treatment and complications.

**Figure 3:**
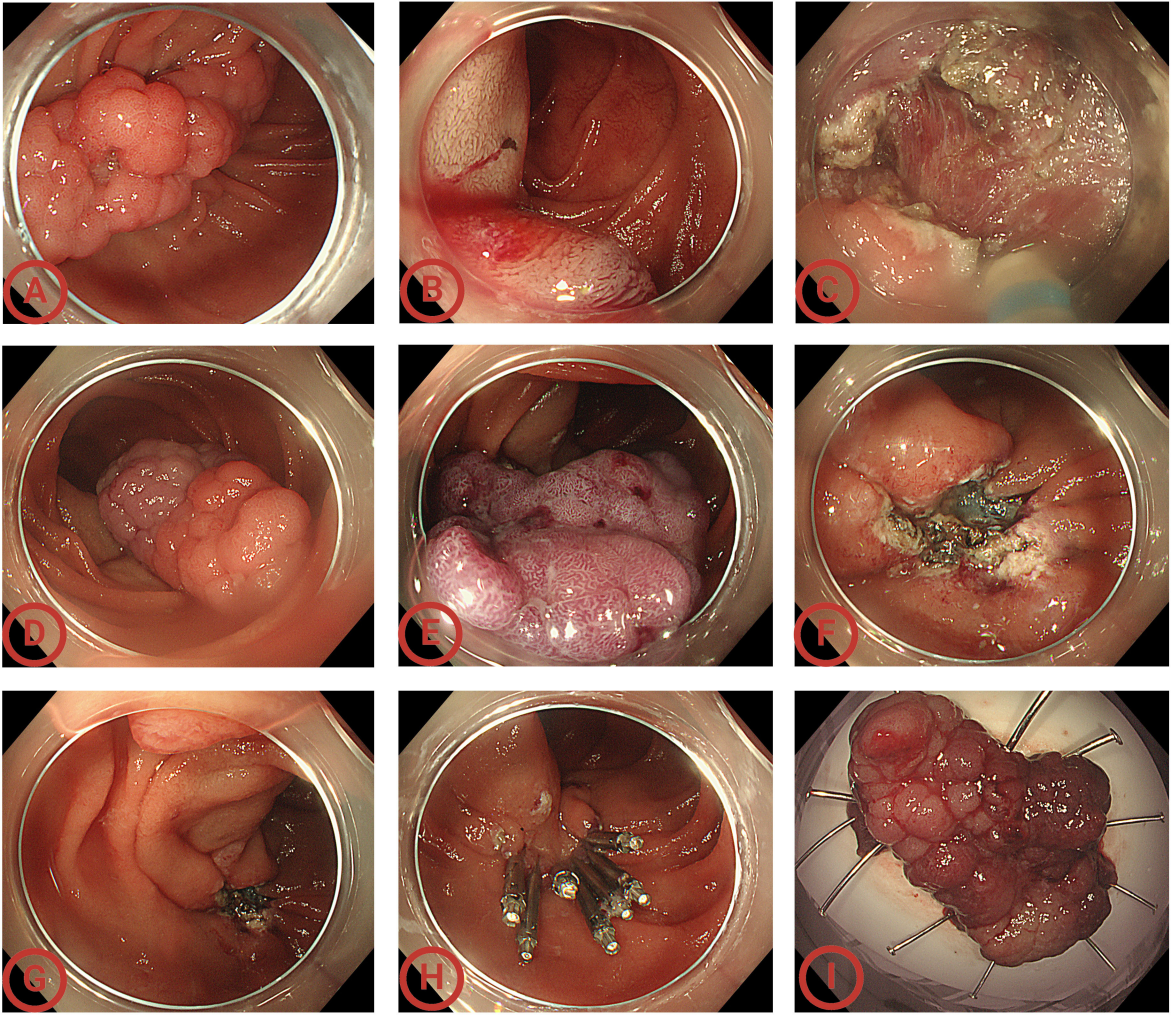
Endoscopic views of the whole operation. **(A)** Endoscopy showed a sporadic non-ampullary duodenal adenoma (SNADA) at least 5cm in size at the descending part of the duodenum; **(B)** submucosal injection on the anal side; **(C)** circumferential incision using the dual knife; **(D)** during the circumferential incision process, the mass’ color changed gradually; **(E)** performing submucosal dissection until the surface of the whole adenoma turned purple; **(F)** the wound surface after dissection; **(G)** confirming the duodenal papilla was intact and located above the wound; **(H)** closing the wound; **(I)** the 4.5 cm × 2.8 cm × 1.0 cm *en bloc* specimen. Considering that labeling easily causes mucosal damage and leakage after submucosal injection, and the adenoma boundary was clear, the lesion was not labeled.

Intraoperatively, the descending duodenum was reached using a colonoscope (Olympus CF-H290I) with a transparent cap, and an approximately 5-cm wide-based lobulated adenoma was seen on the medial wall of the greater curvature. After confirming that the adenoma was located below the duodenal papilla and that the anal side of the adenoma had reached the transition zone between the descending and horizontal parts of the duodenum, a mixture of normal saline (NS), methylene blue, and sodium hyaluronate was injected into the submucosa on the anal side of the adenoma, which showed a positive lifting sign. We the used a dual knife to circumferentially dissect the submucosal side from the anal side of the adenoma until the surface of the whole adenoma turned purple and then switched to snare *en-bloc* resection. Notably, when large blood vessels were exposed during the dissection process, we performed vascular dissection, and then the dual knife was replaced with a hemostatic forceps for preventive electrocoagulation treatment to avoid a massive hemorrhage and achieve the goal of safe and efficient treatment. We then examined the wound and confirmed that the tumor was completely removed, that the wound was intact with no bleeding or perforation, and that the duodenal papilla was intact. The wound was then closed with Boston clips. The whole operation was uneventful and lasted approximately 2 hours (**Figure 3**). The patient safely returned to the ward and was discharged on postoperative day 5.

**Figure 4:**
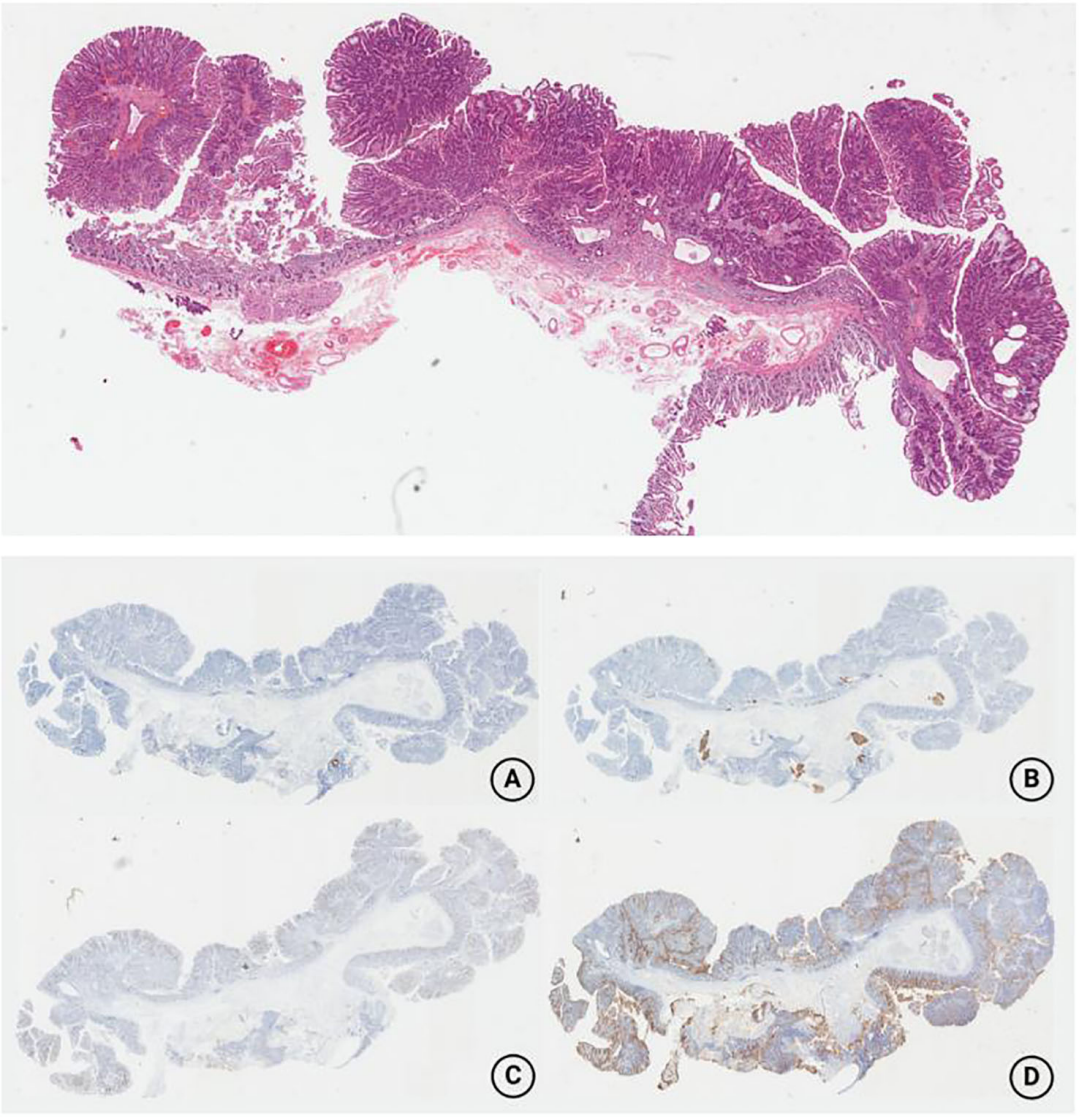
Postoperative pathology revealed the mass to be a tubulovillous adenoma with low-grade intraepithelial neoplasia, a negative base, and a peripheral incisal margin. The immunohistochemistry findings are as follows. **(A)** The MUC5AC expression was negative; **(B)** the MUC6 expression was negative; **(C)** the MUC2 expression was partially positive; **(D)** the CD10 expression was positive.

The completely removed specimen was noted to be 4.5 cm × 2.8 cm × 1.0 cm, which histopathological examinations showed to be a tubulovillous adenoma with low-grade intraepithelial neoplasia, a negative base, and a peripheral incisal margin (**Figure 4**). Further immunohistochemistry exhibited that both MUC5AC and MUC6 expression were negative and MUC2 expression was partially positive, while the expression of CD10 was positive, which indicated an intestinal-type adenoma. The details are shown in **Figure 4**. The patient showed no discomfort during the 1-year follow-up after discharge. Unfortunately, the patient refused further endoscopic reinspection after repeated requests by doctors and family members.

## Discussion

Herein, we described a very rare case of giant SNADA, which was successfully removed using hybrid ESD. Considering that SNADAs have a risk of malignant transformation comparable to colorectal adenomas, we deemed the giant SNADA in this case to warrant intervention, yet few studies have reported related outcomes. Herein, we will further present a case-related discussion that analyzes the case and explores our selection of treatment strategy.

Surgical operation has previously been the main treatment for duodenal adenoma, with complete resection, yet this results in significant trauma and postoperative complications (2). Endoscopic resection is currently the preferred treatment due to the advantages of being minimally invasive, the low incidence of adverse events, and short hospital stay, and the surgery is usually a cure (1, 12). Although EMR is more commonly used, ESD was selected for the giant SNADA in our case (with a lesion with a length of ≥20mm), as it can completely remove the lesion, allow for a complete histological examination, and reduce the risk of recurrence (1). The overall resection rate of ESD is higher than EMR, which is critical since adenomas >2 cm are associated with a higher recurrence rate (13). Furthermore, the peripapillary anatomical structure, especially the vascular structure, is also clearly displayed during the operation, which enables the pre-treatment of vessels and reduces bleeding (14–16). Notably, no recurrence has been observed following an ESD (15, 16). Furthermore, current evidence suggests that hybrid ESD offers advantages over conventional ESD, including easier applicability, shorter procedural times, and fewer adverse events, without compromising *en-bloc* resection rates or increasing recurrence rates, which supports the potential of hybrid ESD as a superior alternative to traditional ESD (19–22).

Based on these considerations, we opted for hybrid ESD for this rare case of giant SNADA to achieve efficient and complete resection while minimizing complications.

However, duodenal ESD requires advanced technology and accuracy. Its operative time is long and the incidence of postoperative complications (mainly hemorrhage and perforation) is relatively high (15), which is mainly due to the following characteristics of the special anatomical structure: (1) the high intraluminal pressure in the gastrointestinal tract leads to thin walls and a high perforation rate; (2) the “C”-shaped configuration and relatively narrow lumen complicate endoscopic maneuvers; (3) abundant submucosal blood supply increase the bleeding risk; (4) the presence of numerous Brunner glands complicates submucosal elevation; (5) proximity to the ampulla and the sharing of a common blood supply with the pancreatic head and the bile pancreatic duct may lead to delayed complications due to postoperative wound exposure to pancreatic fluid and bile, affecting regional blood supply and disrupting biliary-pancreatic duct function (9, 17, 18). Thus special focus should be placed as follows: (1) a colonoscope was preferred given its 6 o’clock orientation when located in the working channel, providing vision for submucosal injection and manipulation while ensuring an adequate endoscopic workspace (18); (2) an adequate submucosal injection is needed to separate the mucosal and muscle layers; (3) we considered that most of the tumor blood supply had been blocked when the tumor turned purple, which means the risk of massive hemorrhage was lower, so we switched from the dual knife to snare resection; (4) the position of the duodenal papilla and the lesion were confirmed repeatedly before and after resection to avoid complications; (5) we balanced the electrocoagulation and electrosection to avoid bleeding and perforation; (6) we ensured that the tumor was completely trapped in the snare (the resection in our case is too large, which may partly blind resection) before the snare resection, and examined the wound after resection to avoid incomplete resection or perforation; (7) ESD-associated mucosal defects may increase the risk of delayed bleeding and perforation, especially given the giant size, and special location (it appeared to be influenced by bile and gastric acid) in our case, thus, the wound was firmly closed to minimize adverse events (23, 24).

Fortunately, with the superb technology and cooperation of our endoscopic team, the giant SNADA was completely removed, with no adverse events and postoperative complications. Subsequent pathology confirmed the giant SNADA to be a tubulovillous adenoma with low-grade intraepithelial neoplasia, which emphasized the necessity for endoscopic treatment, as villous adenomas exhibit higher recurrence rates (25). Additionally, based on the available findings, we recommend a follow-up endoscopy within 3–6 months post-resection to monitor for a recurrence; if not, subsequent endoscopic surveillance at 6–12 months is required (26).

The successful treatment of this case led to the following observations. Due to the difficulty of the operation and serious complications, duodenal ESD should be performed with caution. To establish duodenal ESD as a safe and minimally invasive therapeutic strategy for duodenal tumors, a reliable endoscopic procedure strategy should be developed. Our case suggests that hybrid ESD may be the optimal treatment for duodenal adenomas, but this requires further study. Additionally, although the case reports and guidelines on SNADA are currently deficient, based on our team’s experience, we propose the following options for SNADA: 1) EMR for SNADAs<1cm (offering efficient, safe, and complete resection); 2) for SNADAs 1–2 cm, the choice depends on the morphology of the mass, i.e., EMR for semi-pedunculated or pedunculated masses, and traditional or hybrid ESD for wide-based or flat masses; 3) ESD (traditional or hybrid) for SNADAs >2 cm, especially those >3 cm or with wide base, for complete resection. Generally, SNADA treatment requires individualized evaluation according to the size, location, and shape of the tumor; the doctor’s level of experience with the operation; and the hospital’s multidisciplinary capability to achieve safe, efficient, minimally invasive, and economical treatment. Finally, our research does have certain limitations: 1) Due to the rarity of giant SNADAs, our experience in this field remains limited and the successful resection in this case may have been fortuitous despite the extensive analysis and consideration of treatment strategies; 2) Given the variability in doctors’ levels of experience with the operation and although we considered hybrid ESD to be the most suitable option in this case, there may be more optimized approaches. Consequently, the selection of hybrid ESD in this report represents a sharing of experience from a rare case and should not be considered a universal standard.

## Conclusion

To conclude, we present a very rare case of a giant SNADA that was successfully completely removed by hybrid ESD, which combines both the advantage of ESD (*en-bloc* resection) and snare resection (simple, direct, and fast), enabling the safe, rapid, and complete removal of large duodenal lesions. We believe that hybrid ESD serves as an effective bridge between conventional EMR and traditional ESD, potentially offering a superior or alternative approach to traditional ESD. Furthermore, with advancements in endoscopic technology, hybrid ESD may represent a promising and safe treatment for duodenal lesions, particularly those exceeding 2 cm in size.

## Data Availability

The original contributions presented in the study are included in the article/supplementary material. Further inquiries can be directed to the corresponding author.
